# *Physcomitrium patens CAD1* has distinct roles in growth and resistance to biotic stress

**DOI:** 10.1186/s12870-022-03892-3

**Published:** 2022-11-08

**Authors:** Shan Jiang, Xu Tian, Xiaolong Huang, Jiankang Xin, Huiqing Yan

**Affiliations:** 1 School of Life Sciences, Guizhou Normal University, 550001 Guiyang, China; 2 School of International Education, Guizhou Normal University, 550001 Guiyang, China; 3 Key Laboratory of Plant Physiology and Development Regulation, Guizhou Normal University, 550001 Guiyang, China; 4 Key Laboratory of National Forestry and Grassland Administration on Bioaffiliationersity Conservation in Karst Mountainous Areas of Southwestern China, Guizhou Normal University, 550001 Guiyang, China

**Keywords:** *Physcomitrium patens*, Lignin, Phenylpropanoids, Cinnamyl alcohol dehydrogenase

## Abstract

**Background:**

*Physcomitrium patens* provides an evolutionary link between green algae and vascular plants. Although the genome of *P. patens* includes orthologs of all the core lignin biosynthetic enzymes, the occurrence of lignin in moss is very controversial. Besides, little information is available about the lignin enzymes in moss to date. For example, cinnamyl alcohol dehydrogenase (CAD) is a crucial enzyme that catalyzes the last step of the lignin biosynthetic pathway, suggesting an ideal way to study the evolutionary process. By investigating the functions of *CAD* in evolution, this study will elucidate the evolutionary roles of lignin-like in the early stage of land colonization.

**Results:**

CAD multigene family in *P. patens* is composed of four genes. The PpCADs contain a conserved glycine-rich domain to catalyze NADPH-dependent reduction to their corresponding alcohols, indicating that PpCADs have the potential to synthesize monolignols by bioinformatics analysis. Even though PpCAD1 could produce lignin in theory, no conventional monomer was detected in the cell wall or cytoplasm of *PpCAD1*_OE plants. However, the phenylpropanoids were promoted in *PpCAD1*_OE transformants to modify gametophore architecture and development, making the distribution of phyllids more scarcity and the moss colony more giant, possibly due to the enhanced expression of the *AUX-IAA* family. The transcripts of at least one gene encoding the enzyme in the lignin biosynthetic pathway were increased in *PpCAD1*_OE plants. In addition, the *PpCAD1*_OE gametophore inhibited the *Botrytis cinerea* assault mainly by enhanced phenylpropanoids in the cell wall instead of influencing transcripts of defense genes *pathogenesis-related 10* (*PR10*) and *nonexpresser of PR genes 1* (*NPR1*). Likewise, ectopic expression of *PpCAD1* in *Arabidopsis* led to a significant increase in lignin content, exhibiting chunky roots, robust seedlings, advanced flowering, and efficient resistance against pathogens.

**Conclusion:**

*PpCAD* occurs in more than one copy, suggesting functional divergence in the ancestral plant. *PpCAD1* catalyzes monolignol biosynthesis and has homologous functions with vascular plants. Despite no detected conventional monolignol, the increased phenylpropanoids in the *PpCAD1*_OE gametophore, possibly intermediate metabolites in the lignin pathway, had conserved functions during the evolution of terrestrial plants. The results inferred that the lignin enzyme of the early non-vascular plant played roles in stem elongation and resistance against pathogens of *P. paten****s*** during the conquest of land.

**Supplementary Information:**

The online version contains supplementary material available at 10.1186/s12870-022-03892-3.

## Background

Lignin is a phenolic heteropolymer deposited in the secondary cell walls of vascular plants and plays critical roles in long-distance water transport, mechanical support, and stress response [1]. As a complex and non-linear phenolic polymer, three monolignols of p-guaiacyl (G), hydroxyphenyl (H), and syringyl (S) polymerized lignin to form multiple lignified tissues. The lignin content, monomeric composition, and frequency of intermonomer linkages vary significantly in the different cell types, growth stages, and even in plant evolution [2]. For example, only G lignin exists in ferns, whereas G and a trace amount of H lignin are found in gymnosperms. The presence of S lignin has been restricted solely to angiosperms [3]. In this review, studying the roles of lignin helps to understand plant evolutionary development.

Lignin exerts essential functions in plant development. For example, lignin promotes cell wall rigidity due to the hydrophobic properties of the vascular bundles [4]. The significant decrease of lignin in *Arabidopsis thaliana* and poplar induces stunted growth, fewer branches, and smaller flowers and stems, which might be regulated by mitosis factors and auxin signal pathways, such as kinesin and *Auxin/Indole-3-Acetic Acid* (*AUX/IAA*) [5–7]. Additionally, lignin modification improves fermentable sugar yields [8]. Low molecular weight monomers derived from G and H units have an antioxidant capacity [9]. Importantly, lignin also responds to biotic and abiotic stresses essential in adapting to unfavorable environments [10].

The monolignols start with the amino acid phenylalanine, then are synthesized through a branch of the phenylpropanoid pathway in the cytoplasm. The monolignol was subsequently transported, polymerized, and cross-linked by connecting with cellulose and hemicellulose in the secondary cell wall [11]. Cinnamoyl alcohol dehydrogenases (CAD) is a critical and last enzyme in monolignol biosynthesis that catalyzes the final reaction to synthesize G-, H-, and S-lignin units. *CAD* firstly appeared in some symbiotic fungi and several unicellular algae species early in plant evolution [12]. The functions of CAD have been studied in vascular plants, such as *Arabidopsis*, *Triticum aestivum*, *Oryza sativa*, and *Populus* plants [13, 14].

*CAD* occurs in more than one copy in many species. The divergence of *CAD* may be due to the need for evolutionary development. Some are required for monolignol biosynthesis to influence plant growth and development, whereas others are responsible for adapting to environmental stresses. For example, *AtCAD1* played a substantial role in lignification [15]. Besides, it controlled the programmed cell death by inhibiting expressions of defense genes, such as pathogenesis-related proteins (*PR1* and *PR2*) and NONEXPRESSOR OF PR GENES 1 (*NPR1*) in the salicylic acid signaling pathway [16]. *AtCAD4/5* is also the catalytically most active in synthesizing G/S monolignols [17]. *AtCAD7* lost the lignin biosynthetic activity, but it acted as a negative plant immunity regulator induced by *Phytophthora* infection [18]. Moreover, the transcripts of *CAD* varied in different organs. *AtCAD1* was exclusively expressed in the primary xylem, whereas *AtCAD2/3* was highly expressed in the meristem and root cap but not in lignified tissues. *AtCAD4* was mainly expressed in roots and flowers but low in stem xylem. *AtCAD5* was only highly in roots [19, 20]. To date, the effects of *CAD* on phenotypic traits of transgenic vascular plants have been extensively identified, and *CAD* engineering to modify lignin content or composition is widely used [21, 22]. Nevertheless, little information is available about the roles of CAD in early terrestrial non-vascular plants.

*Physcomitrium patens* acts as the evolutionary link between green algae and angiosperms, allowing an evolutionary analysis during land colonization. *P. patens* can be used to study plant physiology, genetics, and gene functions based on high-frequency homologous recombination [23]. The draft genome sequence is available online (http://www.cosmoss.org) and is constantly being improved [24]. Furthermore, *P. patens* has a simple developmental life cycle with a predominant haploid phase, which dramatically facilitates genetic analysis [24]. In addition, phyllids, rhizoids, and protonemal filaments of *P. patens* consist of only one cell layer, making easy observation using microscopic analysis [25]. Even though the existence of lignin in moss is still controversial, *P. patens* has all the core lignin biosynthetic genes without ferulate 5-hydroxylase (F5H) [9]. Moss is the host for original lignin or contains uncondensed monolignol-like molecules [26]. In addition, a sudden expansion of gene family members about monolignol biosynthesis occurs in *P. patens* compared with the green algae species. Also, *P. patens* is the first species with monolignol biosynthesis gene families, representing the complete monolignol biosynthesis across the plant kingdom [27]. No more than five gene family members of the lignin enzymes exist in any species earlier than *Physcomitrium patens* [9]. Thus, *P. patens*, the basal species for terrestrial plants, can be considered the plant where and when the coordinative evolution of monolignol biosynthesis begins [28]. Therefore, *P. patens* provides an excellent model to elucidate evolution by analyzing *PpCADs* in the early land of colonization.

In the present study, the protein structures, phylogenetic analysis, and expression of *PpCADs* were determined. A detailed functional analysis of *PpCAD1* was explored using knockout and overexpression procedures. Additionally, the increased phenylpropanoids of *PpCAD1*_OE gametophore in the cell wall promoted resistance against *Botrytis cinerea*. Finally, ectopic expression of *PpCAD1* in *Arabidopsis* revealed that *PpCAD1* increased lignin content, regulated plant growth, and enhanced resistance to biotic stress. The results determined the roles of *PpCAD1* in initiating terrestrialization and revealed evolutionary insights of lignin biosynthesis into the conquest of land by the early ancestral plants.

## Materials and methods

### Plant material, pathogen, and inoculation observation

*Physcomitrium patens* was grown on 0.7% agar plates of PpNH_4_ medium (Additional file 1). The plants were cultured at 25 °C with a light cycle of 16 h light/8 h darkness and a light intensity of 55 µmol s^− 1^ m^− 1^. The gametophore of *P. patens* was deposited with permission at the herbarium of Guizhou Normal University (GZNU-PP-01). *B. cinerea* was cultured in Petri dishes on Potato Dextrose Agar (PDA) at 25 °C for 10 days. The spore suspension was obtained by flooding the fungal culture with sterile distilled water, rubbing the mycelium, and filtering through sterile nylon gauze (mesh of 200 μm). The spores of *B. cinerea* were suspended in a PDB medium (PDA medium without agarose). PDB without spores was used as a mock-inoculated treatment. The conidial concentration was adjusted to 5 × 10^6^ conidia mL^− 1^ for electron microscopy and 1 × 10^5^ conidia mL^− 1^ for light microscopy and other procedures [29]. We counted the cells of hyphae infection using a light microscope (BX-61, Olympus, Tokyo) at 0.5, 1-, 2-, and 3-days post-inoculation (dpi) and calculated the rate of hyphae infection as previously reported [29].

### Phylogenetic tree and alignment

Sequences of four putative PpCAD proteins and 26 protein sequences of CAD enzymes from other species were aligned with CLUSTAL W (https://www.genome.jp/tools-bin/clustalw). Conserved motifs were detected by submitting sequences to Pfam (http://pfam.janelia.org). A phylogenic tree was constructed using a maximum-likelihood method in MEGA 10 (https://www.megasoftware.net/). The reliability for the internal branch was assessed by the bootstrap with 1000 bootstrap replicates and marked above the nodes.

### Isolation of RNA, cDNA synthesis, and qRT-PCR analysis

The total RNA of *P. patens* gametophores was extracted using TRIzol Reagent (TaKaRa, Tokyo) according to the manufacturer’s protocol. Then the extracted RNA (2 µg) was reversely transcribed using an RT-PCR Kit® (TaKaRa, Tokyo) with an oligo dT-adaptor primer. Quantitative PCR was performed in a LightCycler®480 instrument (Roche, Switzerland). The reaction mixture consisted of 5 µL FastStart DNA Master SYBR Green I kit (Takara, Tokyo), 0.25 µL each of the gene-specific primers (Additional file 2), 3.5 µL H_2_O, and 1 µL diluted cDNA as template. The cycling procedures were: 95 °C for 10 min, followed by 40 cycles of 95 °C for 20 s, 55 °C for 20 s, and 70 °C for 20 s. The transcripts relative to the control were estimated by calculating ΔΔ Ct and analyzed using the 2^−ΔΔCt^ method. Data were performed three times and showed as means ± standard deviations (SD). The one-way ANOVA was used to determine the statistical significance of differences between the values of the various experimental and control groups, followed by Dunnett’s test. A *p*-value less than 0.05 was considered statistically significant.

### Preparation of protoplast, PEG-mediated plant transformation, and phenotype observation

The *P. patens* protonema cultured for 5–7 days were scraped off cellophane-overlaid agar plates, and about 80 mg of tissue was measured in a 90 mm Petri dish, adding 3 mL of 1% driselase (Sigma D9515-25 G) and incubated with gentle shaking for one hour at room temperature. The protoplast suspension was filtered through a 100 μm mesh, centrifuged at 250 g for 8 min, and re-suspended with 9.5 mL 8% mannitol in the culture tube. A total of 10 µL protoplast was used to count the number by a hemocytometer [21].

A 681-bp and a 650 -bp fragments homologous to the 5’ and 3’ region of *CAD1* were amplified using primers *PpCAD1.1* and *PpCAD1.2*, respectively (Additional file 2). The 5’ and 3’-PCR products were digested via *XhoI*/*EcoRV* and *Xbal*/*BamHI*, ligated into pTN182 to generate *PpCAD1.1*-pTN182-*PpCAD1.2* knockout vector. For a *CAD1* overexpression vector generation, the cDNA was amplified using primers *PpCAD1.3* which contained restriction sites of *EcoRV*/*ApaI*. The volume of ligase was 10 µL prepared with 6 µL target gene fragment, 2 µL digested fragment from pTFH15.3, 1 µL T4-DNA ligase, and 1 µL10*T4 DNA Ligase Buffer. The reaction was performed at 16 ℃ overnight.

Polyethylene glycol (PEG) -mediated transformation of protoplasts was performed as Schaefer et al. described with minor modifications [21]. Briefly, protoplasts were suspended in sterile MMM solution (Additional File 1) at the concentration of 1.6 × 10^6^ protoplasts mL^− 1^. A 30 µg linearized DNA and 300 µL sterile PEG were added to 300 µL protoplast suspension, then placed in the PRM/T medium (Additional File 1). Protoplasts regenerated their cell walls for seven days in a PRM/B medium and were allowed to grow for two weeks on a PpNH4 medium containing 50 µg mL^− 1^ of geneticin (also known as G418) for two weeks. After two rounds of selections, stable moss transformations survived and were testified by PCR using three pairs of primers listed in Additional file 2.

The hidden Markov model (HMM) profile of auxin-responsive genes *AUX/IAA* family (PF02309) and *kinesin* family (PF00225) were retrieved from the *P. patens* genomic database using HMMER3.0 with the cutoff of default parameter set to 0.01, verified with the PFAM and Simple Modular Architecture Research Tool (SMART) web server (http://smart.embl-heidelberg.de/) [30, 31]

### Detection of phenylpropanoids and monolignols in the cell wall

The cell wall was extracted from mock-inoculated plants and *P. patens* at 1, 2, 3, and 5 dpi. The gametophores of *P. patens* were dried at 55 °C for 24 h. The 0.1 g sample was measured and homologized with 4 mL 80% ethanol, ultrasonic for 10 min, and centrifuged at 4,500 rpm for 5 min. The residue was collected and washed with 4 mL of 80% ethanol. The residue was extracted with 4 mL chloroform/methanol (2:1, v/v) two times, and 4 mL acetone was added to remove low-molecular-weight soluble components. The sample was mixed with the acetyl bromide reagent 25% (v/v) acetyl bromide in glacial acetic acid, then at 70 °C for 15 min, quantitatively transferred with the aid of acetic acid to 25 mL volumetric flasks that contained 4.5 mL of 2 M NaOH and 10 mL of acetic acid, 0.5 mL of 0.5 M hydroxylamine. Then the extraction was detected at 280 nm with three biological replicates by spectrophotometer [32].

The cell wall of *P. patens* (60 mg) was treated using the procedure of derivatization followed by the reductive cleavage (DFRC) for gas chromatography-mass spectrometry (GC-MS) analysis [32]. About 80 µL products were loaded onto a solid phase extraction (SPE) column (3 mL silica, normal phase, Supelco, Bellefonte, Pennsylvania) and then eluted with 12 mL cyclohexane/ethyl acetate (5/1, v/v). Procedures for the GC-MS analysis were performed as follows: column, 0.25 mm×30 m SHR5XLB; helium carrier gas, linear-velocity flow-control mode at 35.0 cm/sec; 15:1 split ratio; injector temperature, 250 °C; initial column temperature, 140 °C; temperature ramped at 3 °C/min to 250 °C then ramped at 10 °C/min to 280 °C and held for 1 min, then ramped at 20 °C /min to 300 °C and held for 15 min.

### Detection of phenylpropanoids in the cytoplasm

The gametophores of *P. patens* were fully ground in liquid nitrogen. The sample (0.3 g) was dissolved with 5 mL methanol. After centrifugation (4,000 rpm, 10 min) of the extract, the supernatant was concentrated and rinsed with 4 mL 0.2% acetic acid containing cyclohexane (1:1, v/v). The mixture was subjected to MCI GEL CHP 20P (Sigma-Aldrich) resin solid-phase extraction column, and ethanol was used as eluent. The extraction was filtered with a 0.22 μm ultrafiltration membrane (Pall Corp., Port Washington). Then the analysis was carried out by high-performance liquid chromatography (HPLC) equipped with a photodiode array detector (Agilent 1,200 system). The experiment was under the following conditions: C18 column (4.6 mm×250 mm, 5 μm, Dikma Technologies, Beijing); injection volume, 20 µL; column temperature, 35 °C; flow rate, 1.0 mL/min; solvent system, acetonitrile (A): 0.2% acetic acid (B) with gradient elution. The gradient conditions were 3% A for 6 min, then a linear gradient to 90% over 65 min, and the detection wavelength was 280 nm.

### Safranin-O staining, spectrophotometric measurement of CAD activity and total phenols

The phenolic compounds were detected with safranin-O staining to detect cell modification. The adult and upper phyllids of WT and transgenic plants were incubated with 0.01% safranin-O in 50% ethanol for 5 min. Bright-field microscopy was performed with an Olympus BX61 microscope [33].

A total of 0.2 g gametophores was ground into homogenate and dissolved in a reaction mixture containing 100 mM Tris-HCL (pH 8.8), 20 mM coniferyl alcohol, and 5 mM NADP^+^. CAD activity was measured spectrophotometrically at 450 nm with the kit (Solarbio, Cat#BC4170, Beijing). The reaction was carried out at 30 ℃ and performed as described by Goffner et al. with slight modifications [34]. The phenolic content was determined using the kit (Solarbio, Cat#BC1345, Beijing).

### ***Arabidopsis thaliana*** transformation, identification, and inoculation observation

The seeds of wild-type *Arabidopsis* ecotype Columbia − 0 (Col-0) were germinated on the aseptic MS medium by keeping them in darkness at 4 °C for three days to break dormancy. Then, the seeds were grown in long-day conditions (16 h light/8 h dark) for two weeks. After two weeks, the plants were transferred to soil and maintained in an environment-controlled chamber at 25 °C under the 16 h/8 h light/dark cycle to induce flowering. *PpCAD1* was cloned into the pBI121 vector with *XbaI* and *SmaI* (Additional file 2). Then pBI121-*PpCAD1* vector was transformed into *Agrobacterium tumefaciens* GV3101, and *Arabidopsis* plants were subsequently transformed by the floral dipping method using *Agrobacterium* GV3101 [35].

Phenotypes of transgenic plants at 7 days after germination (DAG) and seedlings were observed in at least three independent lines. The 4-week seedlings of Col-0 and transgenic *Arabidopsis* were inoculated with 10 µL droplets of *B. cinerea*. Trays were covered with lids and kept under the same conditions as for plant growth. The symptoms of seedlings were photographed at 1, 3, and 5 dpi.

### Transmission electron microscopy

The gametophores of *P. patens* were prefixed with 2.5% (v/v) glutaraldehyde in 66.7 mM phosphate buffer, pH 7.4 at 4 °C overnight with *B. cinerea* and then fixed with 1% buffered osmium tetroxide at 25 °C ± 2 °C for 1 h. The fixed gametophores were dehydrated by increasing concentrations of ethanol (30%, 50%, 70%, 80%, 90%, and 100%) at room temperature and embedded in Spurr’s resin (Nisshin EM, Tokyo). Ultrathin sections were cut from these resin blocks with a Porter Blum MT-1 ultramicrotome using a diamond knife (Diatome, Switzerland). The sections were stained with 4% uranyl acetate for 10 min and then with lead citrate for 10 min to observe the infection behavior of *B. cinerea* in the *P. patens* gametophores. For detection of lignin-like substance accumulation in the cell wall of *P. patens*, the sections were stained with 1% solution of KMnO_4_ in 0.1% sodium citrate for 30 min. After being washed with distilled water, they were stained with 2% uranyl acetate for 10 min, and lead citrate for 5 min. Samples were observed with a transmission electron microscope manufactured by Hitachi S-4700 (Hitachi Co., Tokyo).

## Results

### Characteristics of ***P. patens*** CAD genes

**Fig. 1:**
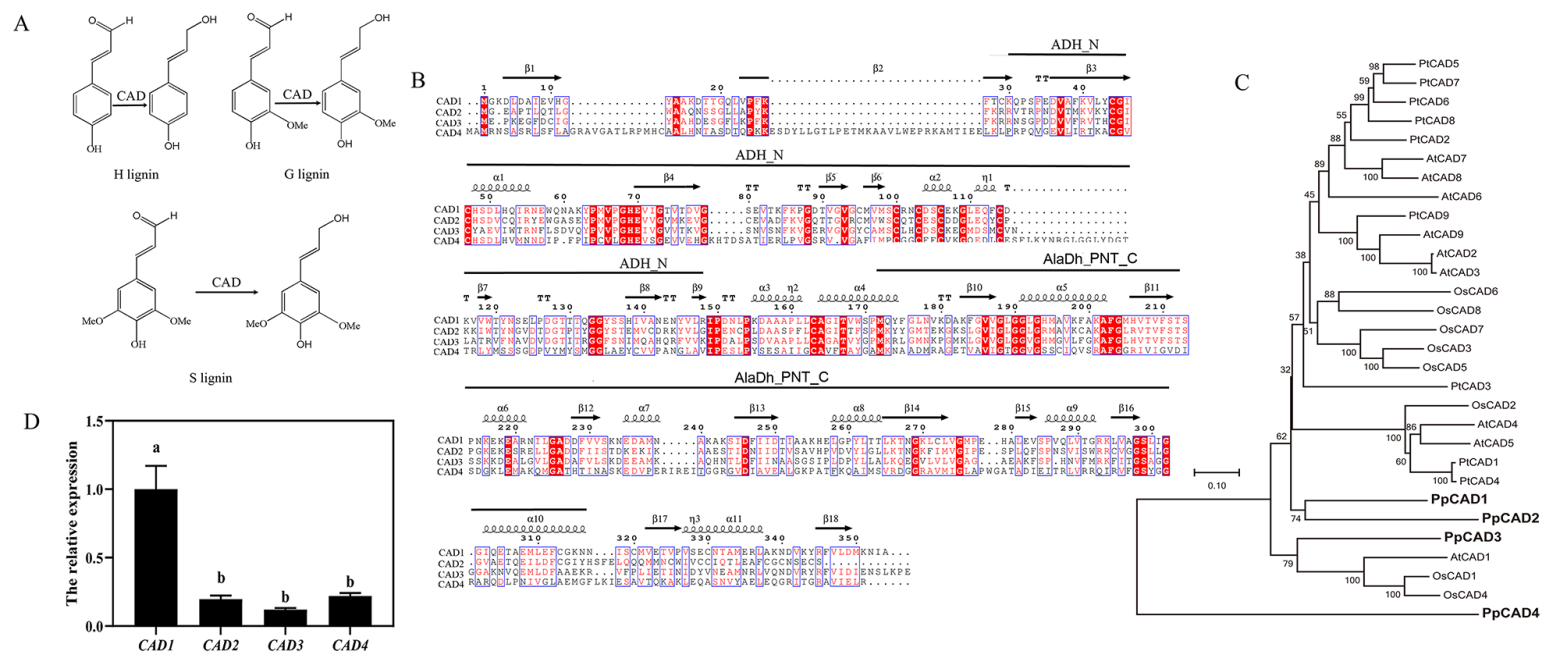
Characterization of PpCADs. (**a**) The function of CAD in vascular plants. (**b**) The domains of PpCADs. (**c**) Phylogenetic analysis of the CAD protein family. Accession number of proteins used to build the tree are: *Arabidopsis thaliana* AtCAD1 (NP_195643); AtCAD2 (NP_195510); AtCAD3(CAA48027); AtCAD4 (NP_195512); AtCAD5 (NP_188576); AtCAD6 (NP_195149); AtCAD7 (NP_179765); AtCAD8 (NP_179780); AtCAD9 (NP_177412); *Populus tremuloides* PtCAD1 (AAF43140); PtCAD2 (AAK58693);PtCAD3 (AAK58693); PtCAD4 (AAK58693); PtCAD5 (AAK58693); PtCAD6 (AAK58693); PtCAD7 (AAK58693); PtCAD8 (AAK58693) PtCAD9 (AAK58693); *Oryza sativa* OsCAD1 (DAA02237); OsCAD2 (DAA02237); OsCAD3 (DAA02237); OsCAD4 (DAA02237); OsCAD5 (DAA02237); OsCAD6 (DAA02237); OsCAD7 (DAA02237); OsCAD8 (DAA02237); OsCAD2 (DAA02237). (**d**) *PpCADs* transcripts in *P. patens* gametophores. Different letters indicated significance in the expression levels at the 0.05 level

CAD catalyzes the final step of syringyl (S), hydroxyphenyl (H), and guaiacyl (G) monolignol biosynthesis in vascular plants (Fig. 1a). Four putative CAD genes were screened based on the genome annotation of *P. patens* and their detailed information was listed in Table 1. Three CAD genes encoded ~ 350 amino acids except for PpCAD4 (425 amino acids). The isoelectric points of PpCAD1 (5.88) and PpCAD4 (7.96) differed markedly. PpCAD1 showed considerably higher identities with PpCAD2, whereas PpCAD1 had only 44% and 21% identity with PpCAD3and PpCAD4, individually.

**Table 1:** Basic information of CAD gene family based on genome

Name	NCBI Accession no.	Accession no. Pytozome	CDS length (bp)	Length (aa)	Molecular weight KDa)	Isoelectric point
*CAD1*	XM_001773423.1	Pp1s163_63	1065	354	38457.30	5.88
*CAD2*	XM_001770443.1	Pp1s126_185	996	331	36011.45	6.03
*CAD3*	XM_001766362.1	Pp1s84_209	1071	356	38395.32	6.51
*CAD4*	XM_024531924.1	Pp1s95_68	1278	425	45398.46	7.96

Upon further investigation of the putative CAD proteins, PpCADs contain various amino acid substitutions, but the four PpCADs are highly conserved. The alignment of PpCADs resulted in the identification of the Zinc-containing ADH_N domain-containing GHE(X)2G(X)5G(X)2 V (68–82 aa), and AlaDh_PNT_C domain composing a glycine-rich repeat V(X)G(X)GG(X)G (186–193 aa) motif involved in NADP(H) cofactor binding of catalytic function, considered as indicative of a characteristic fingerprint for the CAD family. The conserved motifs were marked above the sequences (Fig. 1b). PpCADs are Zn^2+^-dependent alcohol dehydrogenases that belong to the medium-chain dehydrogenase/reductase superfamily

A phylogenetic analysis of 30 deduced amino acid sequences from other species was performed (Fig. 1c), covering monocot and dicot angiosperms. PpCAD1 fall within the same branch as PpCAD2. PpCAD3 was closely related to AtCAD1 and OsCAD1/4. However, PpCAD4 was separated into a distinct branch, inferring that PpCAD4 participated in the evolutionary divergence of flowering and non-flowering plants

We further explored the expression analysis of *PpCAD* genes in gametophores (Fig. 1d). The results showed that the expression of *PpCAD1* was almost 5-fold compared with others. However, the transcript of *PpCAD2*, *PpCAD3*, or *PpCAD4* displayed low levels in *P. patens*, and there was no noticeable difference between the three genes. Therefore, PpCAD1 might play a critical role in the growth and development of *P. patens*, and we made further investigations

### ***PpCAD1*** influenced the gametophore architecture and biomass

**Fig. 2:**
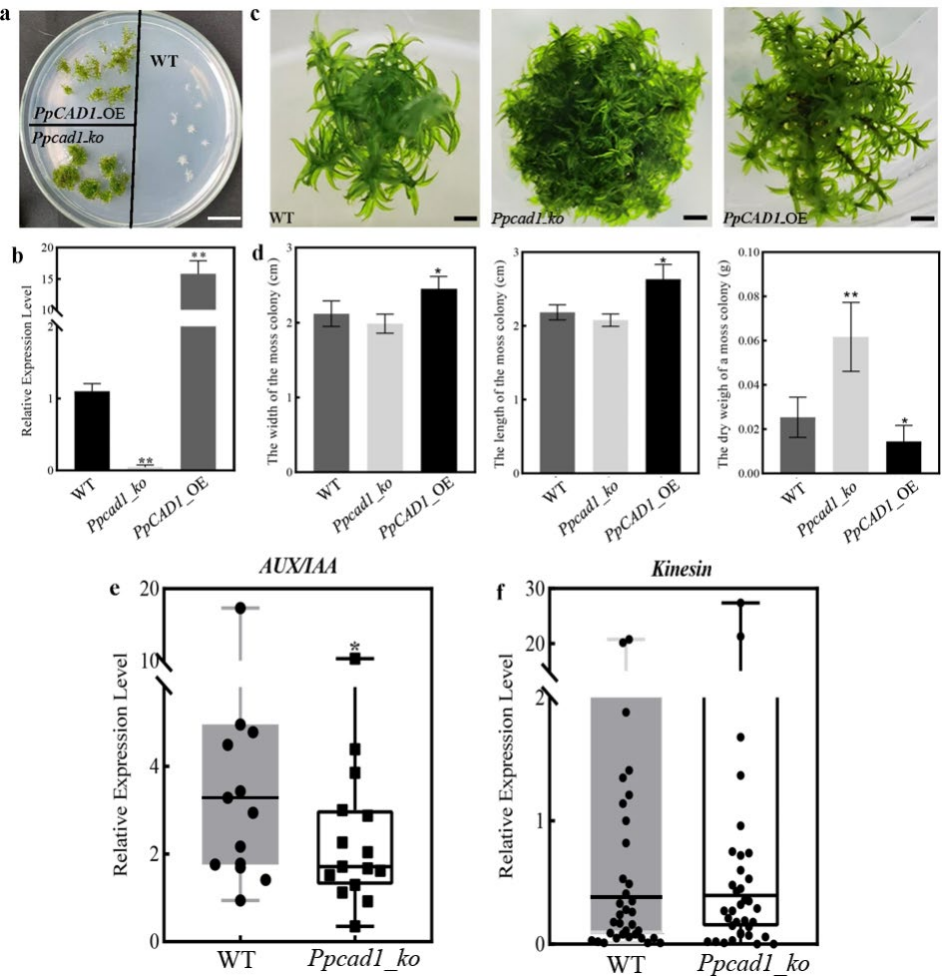
The phenotypes of transgenic gametophores. (**a**) The G418 resistant selection (bar = 1 cm). (**b**) *PpCAD* transcripts in three genotypes. (**c**) Architectures of three genotypes (bar = 2 mm). (**d**) The width, length, and dry weight of each moss colony. The transcriptional distributions of (**e**) *AUX/IAA* family and (**f**) *kinesin* gene family in WT and *Ppcad1_ko* transformants. Values were presented as means ± SD and analyzed using one-way ANOVA (**p < 0.01, *p < 0.05)

To gain more insight into the roles of *PpCAD1* in moss, we generated *PpCAD1* knockout (Additional file 3) and overexpression transformants (Additional file 4). Resistant selection and PCR verification assessed the stable transformants. By homologous recombination, *Ppcad1_ko* and *PpCAD1*_OE transformants survived the PpNH4 medium with 50 µg/mL G418 after two rounds of selections, whereas wild-type (WT) gametophore died, indicating the resistant gene was efficiently integrated into the moss genome (Fig. 2a). Furthermore, the qPCR analysis showed that the expression values of *CAD1* in *Ppcad1_ko* and *PpCAD1*_OE transformants were nearly 0.07 and 15.83 folds compared with WT, respectively (Fig. 2b).

The morphological characteristics during gametophytic growth were altered among the three genotypes (Fig. 2c). *PpCAD1*_OE exhibited loose architecture with a longer stem, whereas *Ppcad1_ko* displayed more tightly and fulfilled with clustered phyllids with spindle orientation, illustrating that *PpCAD1* modified the gametophore architecture by elongating the stem and influencing phyllids distribution. We further measured the width and length of moss colonies using more than 20 transformants of each genotype. Consistently, the width and length of *PpCAD1*_OE colonies were remarkably larger than the others. Interestingly, the dry weight of *Ppcad1_ko* lines bearing more phyllids showed the highest (Fig. 2d). The results revealed that *PpCAD1* positively influenced the elongation of the stem to keep the upright stature of *P. patens*.

Considering that auxin and kinesin are responsible for the growth of *P. patens*, we determined the transcripts of the *Aux/IAA* and *kinesin* gene family to explore whether these genes were modified in response to *PpCAD1* alteration. Fifteen *PpAux/IAAs* and forty-four *Ppkinesins* members were identified based on the *P. patens* genome (Additional file 5). The qRT-PCR analysis revealed that the transcripts of the majority *PpAux/IAAs* accumulated higher in WT compared with *Ppcad1_ko* lines, thereby indicating that *PpCAD1* significantly enhanced the *PpAux/IAAs* levels (Fig. 2e). The expressions of *Ppkinesins* were also altered in *Ppcad1_ko* lines. However, no substantial change of the *Ppkinesins* family was found (Fig. 2f).

### ***PpCAD1*** promoted phenylpropanoids accumulation in the early terrestrial plant

**Fig. 3:**
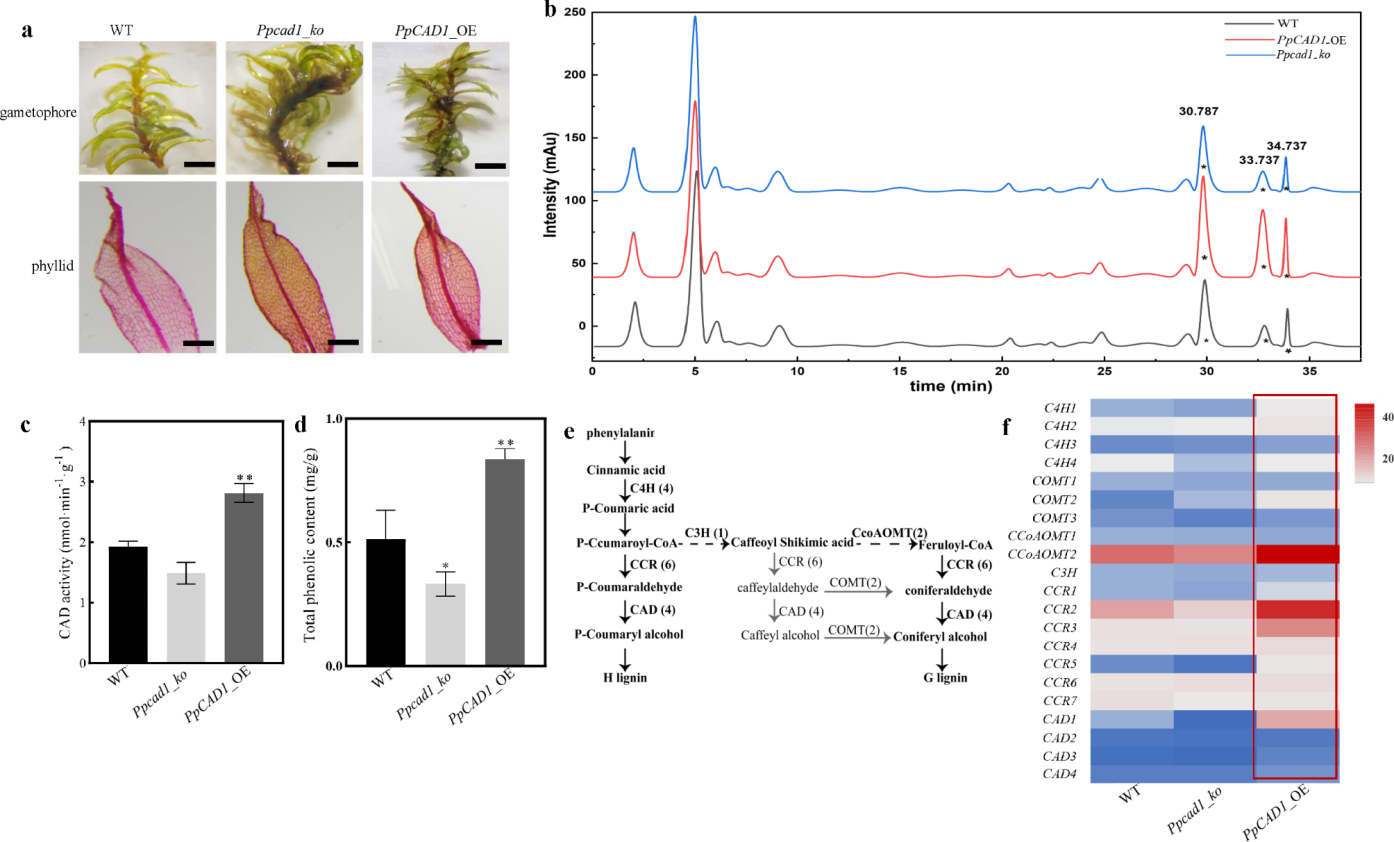
Phenylpropanoids were produced by *PpCAD1*. (**a**) Safranin-O staining of gametophores (bar = 2 mm) and phyllids (bar = 200 μm). (**b**) HPLC fingerprints of extracted cytoplasm from WT, *Ppcad1_ko*, and *PpCAD1*_OE plants. Asterisk indicated significant changes of three peaks. (**c**) CAD activity. (**d**) Total phenolic content. Values were presented as means ± SD and analyzed using one-way ANOVA (**p < 0.01, *p < 0.05). (**e**) Proposed lignin biosynthetic pathway in *P. patens*. The numbers in the bracket represented the number of enzymes in each family. cinnamate 4-hydroxylases (C4H); cinnamoyl-CoA reductases (CCR); cinnamyl alcohol dehydrogenases (CAD); caffeic acid O-methyltransferase (COMT); caffeoyl coenzyme acid O-methyltransferase (CCoAOMT); cinnamate 3-hydroxylases (C3H). (**f**) The expression of genes associated with lignin. The red box indicated a remarkable increase of genes in *PpCAD1*_OE plants. The color scale represented relative expression levels

Apart from morphological alteration, the safranin-O staining signals of *PpCAD1*_OE and WT were more evident than *Ppcad1_ko* gametophore (Fig. 3a). Likewise, more signals in the signal cell layer of phyllid of *PpCAD1*_OE lines and WT were detected, especially in the cell wall, suggesting that more phenols were accumulated in *PpCAD1*_OE and WT plants. To characterize the enhanced substances influenced by *PpCAD1*, three types of lignin units were investigated firstly in the cell wall of *P. patens* by GC-MS according to the roles of the CAD enzyme in generating three monomers in the vascular plant (Additional file 6). First, G and S monolignols were found in bamboo willow (*Salix salicaceae*) using DFRC, and only a small amount of H lignin existed in *Arabidopsis*. However, any of G, S, or H lignin could not be detected in the cell walls of *P. patens*, even in *PpCAD1*_OE transformants.

We attempt to detect the altered compounds of cytoplasm where the monolignols were firstly produced, despite the absence of three monolignols in the cell wall. Three peaks (asterisk) revealed significant changes in the cytoplasm of *PpCAD1*_OE, *Ppcad1_ko*, and WT plants by HPLC (Fig. 3b). The three altered peak areas of *PpCAD1*_OE lines were the most prominent, indicating that *PpCAD1* promoted some kinds of compounds in the cytoplasm. Then three peaks were further scanned by full-wavelength and exhibited strong absorbance values at 263.6 nm, 298.1 nm, and 262.5 nm, individually (Additional files 7 and 8). Since their absorbance values were nearly 280 nm which was the typical absorbance value of benzenoid compounds, the increased molecules in the cytoplasm were predicted to be phenols.

Despite the absence of lignin monomers in the cell wall and cytoplasm, the highest CAD enzyme activity in *PpCAD1*_OE lines was up to 2.82 nmol/min/g, which increased by 43.56% of WT (Fig. 3c). The content of total phenols revealed a tremendous increase in *PpCAD1*_OE lines, which was 1.66 and 2.13-fold compared with WT and *Ppcad1_ko*, respectively (Fig. 3d). All lines of evidence could be indicative of phenols accumulated by *PpCAD1*. The genes involved in the monolignol pathway were investigated to determine whether the phenols were increased through lignin biosynthesis. The proposed lignin biosynthetic pathway in *P. Patens* was simply displayed in Fig. 3e. The diagram illustrated enzymes in the lignin biosynthesis encoded by a gene family. All transcripts of 21 genes were investigated by qRT-PCR (Additional file 9). Except for no significant change in *cinnamate 3-hydroxylases* (*C3H*) transcript, at least one gene encoded each enzyme was substantially increased in *PpCAD1*_OE lines, especially changes of *cinnamate 4-hydroxylases 2* (*C4H2*, 2.68-fold), *caffeic acid O-methyltransferase 2* (*COMT2*, 5.62-fold), *caffeoyl coenzyme acid O-methyltransferase 2* (*CcoAOMT2*, 1.63-fold) and *cinnamoyl-CoA reductases 2* (*CCR2*, 4.7-fold) up-regulated of WT. Overall, a remarkable increase of genes associated with the lignin pathway was detected by incorporating the expression levels of 21 genes (Fig. 3f). We deduced that *PpCAD1* enhanced the phenylpropanoids by promoting the expressions of lignin biosynthetic genes.

### The increased resistance of ***PpCAD1***_OE to ***Botrytis cinerea*** was attributed to the enhanced phenylpropanoids in the cell wall

**Fig. 4:**
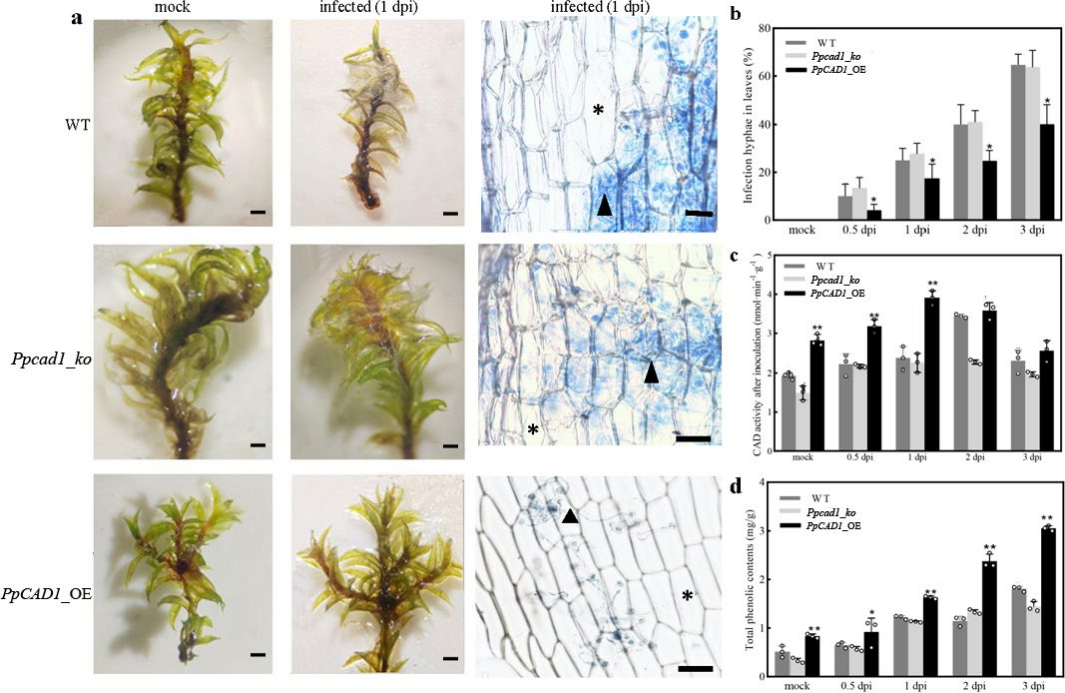
Overexpression of *PpCAD1* efficiently inhibited hyphae infection. (**a**) Phenotype analysis (bar = 1 mm) and histology observation after *B. cinerea* attack (bar = 50 μm) at 1 dpi. Asterisks indicated uninfected cells, and triangles indicated invasive mycelium. (**b**) Invasive hyphae in leaves at 0.5, 1, 2, and 3 dpi. (**c**) CAD activity, and (**d**) total phenolic contents in WT, *Ppcad1_ko*, and *PpCAD1*_OE transformants. All data are presented as means ± SD and analyzed using one-way ANOVA (**p < 0.01, *p < 0.05)

In order to assess the roles of *PpCAD1* against pathogens, the moss colonies of three genotypes were inoculated with *B. cinerea*. (Fig. 4a). At 1 dpi, brown lesions usually progressed from the base to the whole plant. However, the gametophores of WT and *Ppcad1_ko* were covered with mycelium and exhibited blight symptoms, whereas the *PpCAD1*_OE plant displayed higher resistance to *B. cinerea* at the same inoculated time. Most spores germinated laterally and produced germ tubes on the surface of *P. patents*, with the tips of the germ tubes differentiating into an appressorium. We could precisely assess the condition of pathogen infection by observing and counting invasive hyphae. The number of hyphae-infected cells increased gradually with inoculation time. Interestingly, no significant changes in hyphae-infected were between the *Ppcad1_ko* and WT at 1, 2, and 3 dpi (Fig. 4b). However, the number of hyphae-infected cells in the *PpCAD1*_OE was less than in others, thus inferring that *PpCAD1* efficiently restrained pathogen invasion.

After being inoculated with *B. cinerea*, the activities of CAD enzymes were increased to different degrees, especially in *PpCAD1*_OE plants, indicating that the CAD enzyme might be involved in the defense response to *B. cinerea* (Fig. 4c). The activity of CAD enzyme in *PpCAD1*_OE plants was always more prominent than the others during the same inoculated time. Similarly, the total phenols in the three genotypes were increased (Fig. 4d). Likewise, the phenols content in *PpCAD1*_OE transformants was remarkably higher than in the *Ppcad1_ko* and WT.

**Fig. 5:**
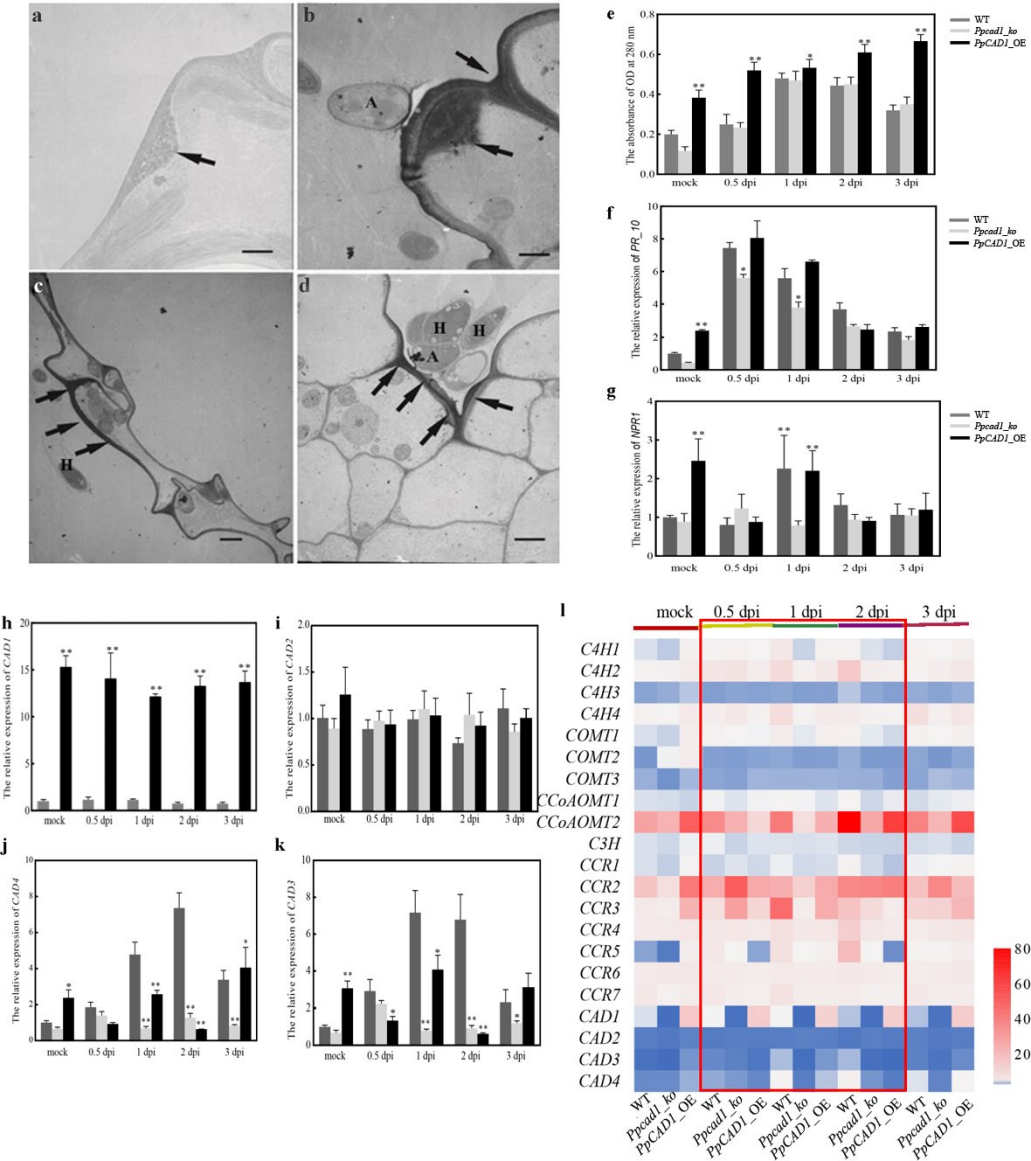
Enhanced phenylpropanoids by *PpCAD1* in the cell wall increased resistance to pathogens. (**a**) Papillae (arrow) were generated in cell walls and cell corners without KMnO_4_ staining as the control. (**b**) The large papillae (arrow) and cell corners of *PpCAD1*_OE lines near the appressorium were stained. Thicker cell walls (arrows) of (**c**) WT and (**d**) *Ppcad1_ko* next to the invasive hyphae showed the high electron density in phyllid. A, appressorium, H, invasive hyphae. Bar = 0.5 μm (**a** and **b**), Bar = 1 μm (**c** and **d**). (**e**) Detection of phenylpropanoids in the cell wall. The transcripts of (**f**) pathogenesis-related 10 (*PR10*), (**g**) nonexpresser of PR genes 1 (*NPR1*), (**h**) *PpCAD1*, (**i**) *PpCAD2*, (**j**) *PpCAD3*, and (**k**) *PpCAD4.* All data are presented as means ± SD and analyzed using one-way ANOVA (**p < 0.01, *p < 0.05). (**l**) Transcripts of 21 genes in three genotypes involved in lignin biosynthesis at 0.5, 1, 2, and 3 dpi. The color scale represented relative expression levels

The electron density of the altered cell walls in unstained sections was lucent when compared with KMnO_4_-stained sections, regardless of papillae, thickened cell walls, or unaltered cell walls (Fig. 5a). The permanganate anion is reduced to manganese dioxide by phenols using KMnO_4_ staining, which precipitates as electron opaque sediments. The papillae or thickened cell walls next to the infection sites of the appressoria or infected hyphae showed an increased electron density compared with other regions during interactions of *P. patens* with *B. cinerea*. We found that papillae induced post *B. cinerea* inoculation in cell walls of *PpCAD1*_OE (Fig. 5b) was more prominent than WT (Fig. 5c) and *Ppcad1_ko* (Fig. 5d). The cell wall extracts of control plants and *B. cinerea*-inoculated *P. patens* were measured at 280 nm (Fig. 5e). The absorbance value of the inoculated plant in *PpCAD1*_OE plants was higher than that in *Ppcad1_ko* and WT, and the difference peaked at 3 dpi. Additionally, *PpPR10* and *PpNPR1* were altered after pathogen assault in each genotype. However, there were no significant changes among the three genotypes (Fig. 5f and g).

We evaluated the *PpCAD* expression to identify the roles in pathogen attacks. Interestingly, *PpCAD1* and *PpCAD2* exhibited no alteration after *B. cinerea*-inoculated compared with mock (Fig. 5 h and i), whereas *B. cinerea* substantially promoted the transcripts of *PpCAD3* and *PpCAD4* (Fig. 5j and k). Besides, the transcripts of 21 genes involved in the monolignol biosynthesis were almost enhanced in *PpCAD1*_OE lines at 0.5, 1, and 2 dpi, deducing that phenylpropanoids, possible intermediate metabolites in the lignin pathway, were increased after *B. cinerea* infection (Fig. 5 L). Thus, the promoted resistance of *PpCAD1*_OE plants to *B. cinerea* was attributed to the increased phenylpropanoids in the cell wall.

### ***PpCAD1*** had a conserved function homologous to vascular plants

**Fig. 6:**
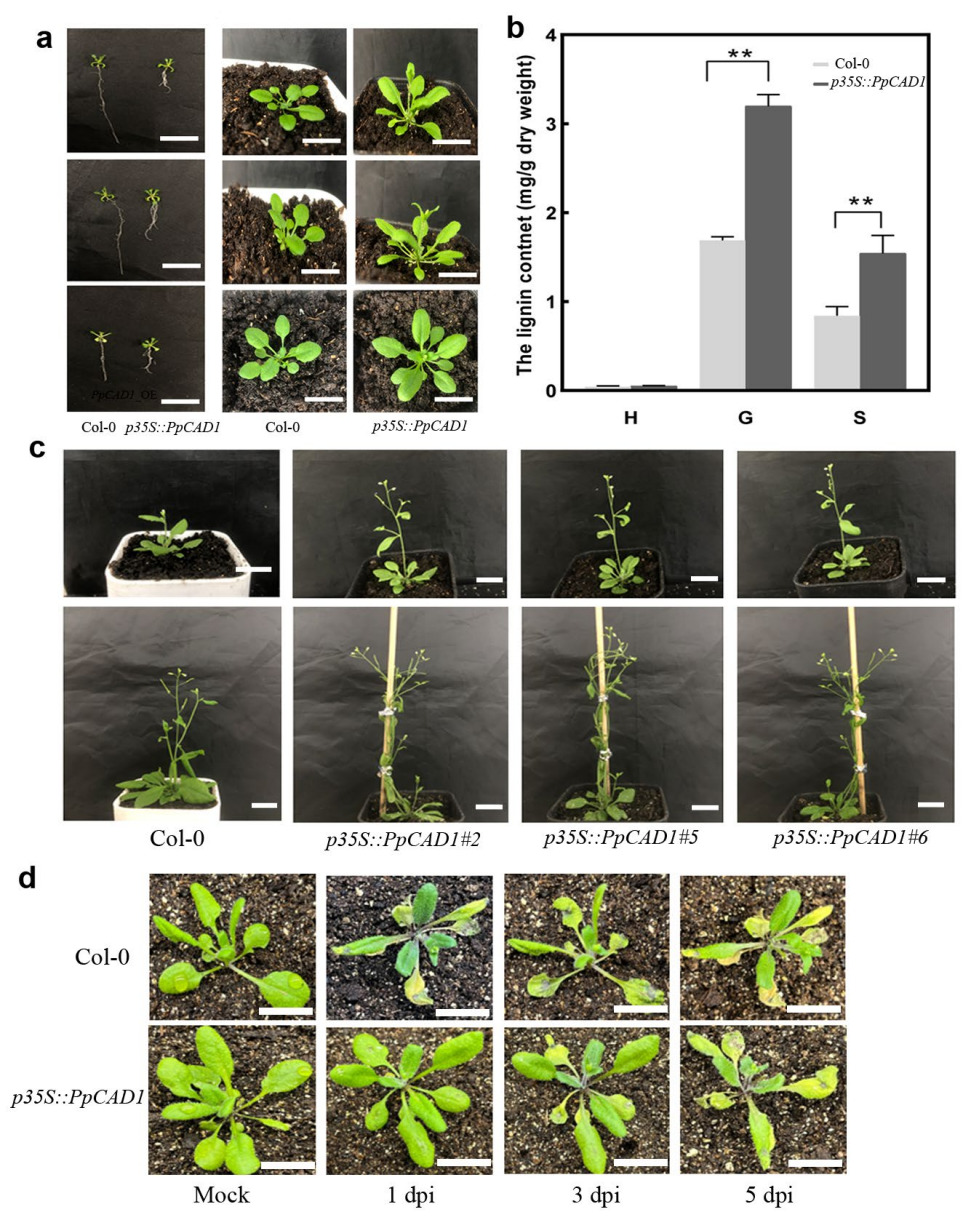
*PpCAD1* increased lignin content, modified plant growth, and enhanced resistance to biotic stress. (**a**) Roots (bar = 1 cm) at 7 DAG and seedlings in Col-0 and *p35s::PpCAD1 Arabidopsis* (bar = 2 cm). (**b**) The content of three monomers. The monolignol content was presented as means ± SD and analyzed using one-way ANOVA (**p < 0.01, *p < 0.05). (**c**) The flowering time of *p35S::PpCAD1* plants was advanced compared with Col-0 (bar = 1.5 cm). (**d**) The plant symptoms of 4-week seedlings of *Arabidopsis* after *B. cinerea* infection at 1, 3, and 5 dpi were observed (bar = 2 cm)

Further ectopic expression of *PpCAD1* in *Arabidopsis* was performed. Apparent plant phenotype alterations were observed between Col-0 and *p35S::PpCAD1 Arabidopsis.* The presence of *PpCAD1* has an impact on dramatic inhibition in root growth and additional emergence of lateral roots near the crown of 7 DAG seedlings *Arabidopsis* (Fig. 6a). The seedlings of *p35S::PpCAD1* plants were more robust than Col-0 during the same growth condition. To determine whether higher expression of P*pCAD1* in the transgenic *Arabidopsis* was correlated with a modified lignin amount or composition, three monomers were detected by GC-MS. Strikingly, the contents of G and S lignin in the overexpression transgenic plants were 3. 20 mg/g and 1.54 mg/g, up to 1.88 and 1.83 times increase of Col-0 individually (Fig. 6b). The results indicated that P*pCAD1* catalyzed monolignol biosynthesis in the vascular plant. Furthermore, the flowering time of *p35S::PpCAD1* plants was slightly advanced compared with Col-0 (Fig. 6c). *PpCAD1* affected plant growth and development, demonstrating that *PpCAD1* could influence plant development homologous to *P. patens* by influencing the lignin content.

The 4-week seedlings of Col-0 and *p35S::PpCAD1 Arabidopsis* were inoculated with a suspension of *B. cinerea* spores or mock-inoculated. Expanding lesions developed on the pathogen-inoculated leaves at 1 dpi (Fig. 6d). However, the brown lesions and blight symptoms of Col-0 were evident at 3 dpi, whereas only brown spots of *p35S::PpCAD1* plants were detected simultaneously. Furthermore, the leaves of Col-0 displayed substantially larger brown areas and more maceration tissues than *p35S::PpCAD1* plants at 5 dpi, suggesting *PpCAD1* enhanced the resistance to *B. cinerea* in *Arabidopsis*.

## Discussion

### PpCAD is homologous to vascular plants and can biosynthesize the monolignols

The occurrence of lignin in moss is very controversial. However, the genome of *P. patens* contains orthologs of all the core lignin biosynthetic enzymes. Among the enzymes, four CAD homologs genes belong to a multigene medium-chain dehydrogenase/ reductase family in *P. patens*. All four proteins have domains of Zn^2+^ dependent ADH_N and AlaDh_PNT_C that are catalytic for alcohol dehydrogenases and NAD-dependent reversible reductive amination of pyruvate into alanine that is the same as AtCAD4/5 [17]. Besides, PpCADs possess a glycine-rich region V(X)G(X)GG(X)G(X). The similar glycine-rich repeat sequences G(X)GGV(L)G also appear in *Populus tomentosa* [36]. The conserved glycine-rich domain involved in NADP(H) cofactor binding includes NADP-dependent quinone oxidoreductase, suggesting that PpCADs have the potential to catalyze the NADPH-dependent reduction of coniferaldehyde, sinapaldehyde, and caffeyl aldehyde to their respective alcohols [37]. Besides, the results proved that the effects of *PpCAD1* in overexpression in *Arabidopsis* caused a remarkable increase of G and S content, leading to a significant increase in lignin content while exhibiting chunky roots, robust seedlings, and advanced flowering. The same phenotypic effects could be modified by another critical enzyme involved in monolignol biosynthesis [38]. Moreover, the lignin-like in *P. patens* and lignin in *Arabidopsis* produced by *PpCAD1* affected plant development. Therefore, PpCAD1 has the capacity to catalyze monolignol biosynthesis according to conserved domains and homologous functions.

### The lignin-like molecules were enhanced by ***PpCAD1*** in ***P. patens***.

Since PpCAD is predicted to have the potential to catalyze monolignol biosynthesis, it is believed that moss evolves different well-bond phenylpropanoids that resemble lignin [39]. There are no non-conventional types of monolignol structure detected in *PpCAD1*_OE gametophores. Despite lignin-like polymers considered B-ring flavonoids similar to H-, G-, and S- lignin units in moss as UV screens, it is believed that the enhanced lignin-like polymers might be intermediate in the lignin biosynthetic pathway [25]. Furthermore, three pieces of evidence support that lignin-like molecules are enhanced in *P. patens*. First, CAD activity and total phenolic content were all enhanced in *PpCAD1*_OE gametophores. Secondly, at least one gene of each family encoding enzymes implicated in the lignin biosynthetic pathway was substantially increased in *PpCAD1*_OE transformants. Thirdly, lignin-like could be deposited at the cell walls of *P. patens* after pathogen inoculation using the potassium permanganate staining procedure at the ultrastructural level. This staining procedure is usually used for localizing lignin compounds in studying the lignification of the vascular plant cell walls [40]. Taken together, *PpCAD1* showed a relation with lignin-like accumulation and played roles in growth and resistance against biotic stress in ancestral plants.

*Aux/IAA* genes could promote lignin to reinforce cell walls [41]. Lignin also regulates moss colony size, gametophore formation, and spindle growth^42^,^43^. Considering the presence of lignin-like in moss, *PpCAD1* significantly increased the expression levels of the majority auxin-responsive genes *AUX/IAA*, thereby displaying more upright stature of *P. patens*. Therefore, the results suggested that *PpCAD1* is responsive to the auxin signaling pathway to elongate the moss.

### Functional divergence of four ***PpCAD*** genes

*PpCADs* had more than one copy and showed different expression profiles in *P. patens*, indicating possibly functional divergence in the early land colonization. The phylogenetic analysis showed *PpCAD1* and *PpCAD2* clustered in the same branch, suggesting the homologous and redundancy functions of *PpCAD2* shared with *PpCAD1*. Consistently, their expressions both displayed no noticeable change after *B. cinerea* inoculation. *PpCAD1* transcripts significantly accumulated in gametophores, indicating that *PpCAD1* might play crucial roles in moss growth. Additionally, we clearly distinguished the morphological alteration by gene manipulation of *PpCAD1*, which made the distribution of phyllids more scarcely and the moss colony more giant. The lignin-like produced by *PpCAD1* in moss is not required to form vascular bundles to facilitate the long-distance transportation of water and nutrients, but it plays a role in supporting upright stature by elongation stem [11]. In contrast, transcripts of *PpCAD3* and *PpCAD4* were remarkably enhanced after the pathogen attack, indicating that *PpCAD3* and *PpCAD4* were mainly involved in defense response.

The phenylpropanoids produced by *PpCAD1* have resistance roles against pathogens in *P. patens*, but pathogens did not cause a significant change in *PpCAD1* transcripts. Therefore, we speculated that *PpCAD1* might not directly regulate the defense response, supported by the no significant changes of defense genes *PR10* and *NPR1* in three genotypes after pathogen infection (Fig. 5). Nevertheless, the CAD activity, total phenolic and lignin-like molecules in the cell wall were significantly enhanced after *B. cinerea* assault, especially in *PpCAD1*_OE gametophores. Therefore, we presumed that the monolignol biosynthesis pathway might be activated in *P. patens* inoculated with *B. cinerea*. Interestingly, *Ppcad1_ko* lines exhibited the same resistant capacity as the WT, mainly contributing to the increased expressions of *PpCAD3* and *PpCAD4*, which were responsible for the compensation of phenylpropanoid generation [44].

*PpCADs* have the potential to catalyze monolignol biosynthesis in ancestral plants and homologous functions with vascular plants in plant terrestrialization. Though no conventional monomer and vascular bundle were detected in *P. patens*, the lignin-like molecules were enhanced by *PpCAD1* that played roles in mechanical support to upright landing, stem elongation, and resistance against pathogens.

## Electronic supplementary material

Below is the link to the electronic supplementary material.

Supplementary Material 1

Supplementary Material 2

Supplementary Material 3

Supplementary Material 4

Supplementary Material 5

Supplementary Material 6

Supplementary Material 7

Supplementary Material 8

Supplementary Material 9

## Data Availability

The datasets generated during the current study are available in the NCBI (https://www.ncbi.nlm.nih.gov/), *PpCAD1* (GenBank number XM_001773423.1), *PpCAD2* (GenBank number XM_001770443.1), *PpCAD3* (GenBank number XM_001766362.1), and *PpCAD4* (GenBank number XM_024531924.1).
