# Supplementary material for: Physcomitrium patens CAD1 has distinct roles in growth and resistance to biotic stress
Source: BMC Plant Biol. 2022 Nov 8;22:518. doi: 10.1186/s12870-022-03892-3 (PMC9641914; doi:10.1186/s12870-022-03892-3)

Additional file 3 The construction of *CAD1* knockout vector and PCR confirmation. **a** The strategy of targeted gene disruption by homologous recombination to knockout gene. Three primers (*P1*, *P2* and *P3*) were used to identify the transgenic plants. **b** DNA extracted from *P. patens* (Lane 1 and 2). **c** The pTN182 plasmid (Lane 1 and 2). The linear and circle types were about 5000 bp and 2500 bp. **d** The *CAD1* knockout confirmation. Lane (1) pTN182; (2) *PpCAD1.1*-pTN182; (3) *PpCAD1.1*-pTN182- *PpCAD1.2*; (4) The constructed vector was treated with *XhoI* and *BamHI*. **e** The PCR confirmation of transgenic plants. Lane (1) WT using primer *P1* to obtain the 2078 bp fragment; Transgenic *P. patens* using *P3*, P1 and P2 to obtain (2) 2197 bp, (3) 4048 bp and (4) 1654 bp fragment. DL 15 000 DNA marker.

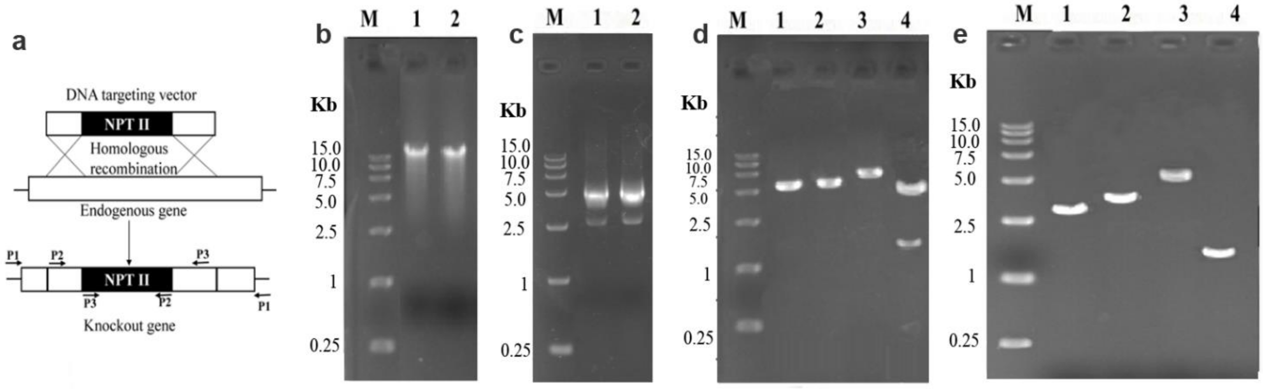

Supplement: Supplementary file 3 — Supplementary Material 3 [file 12870_2022_3892_MOESM3_ESM.pdf]
