# Supplementary material for: Physcomitrium patens CAD1 has distinct roles in growth and resistance to biotic stress
Source: BMC Plant Biol. 2022 Nov 8;22:518. doi: 10.1186/s12870-022-03892-3 (PMC9641914; doi:10.1186/s12870-022-03892-3)

Additional file 4 The generation of overexpression *PpCAD1*-pTFH15.3 and PCR confirmation. **a** The structure of plasmid pTFH15.3, HB7 (Homeodomain 7). **b** The cDNA fragment of *CAD1* (Lane 1 and 2). **c** The confirmation of constructed vector *PpCAD1.1*-pTFH15 was treated with *EcoRV* and *ApaI*. (Lane 1 and 2). **d** The amplification with three primers in wild type. Lane (1 and 2) *Pp15.3-up*; (3) *Pp15.3-down*; (4) *Pp HB7-F*, suggesting that *HB7* was expressed with no disruption. **e** The amplification of *CAD1* overexpression plants with three primers. (1) *Pp HB7-F*; (2) *Pp15.3-up*; (3) *Pp15.3-down*, suggesting that *HB7* was disrupted and *CAD1* was inserted into *P*. *patens*.

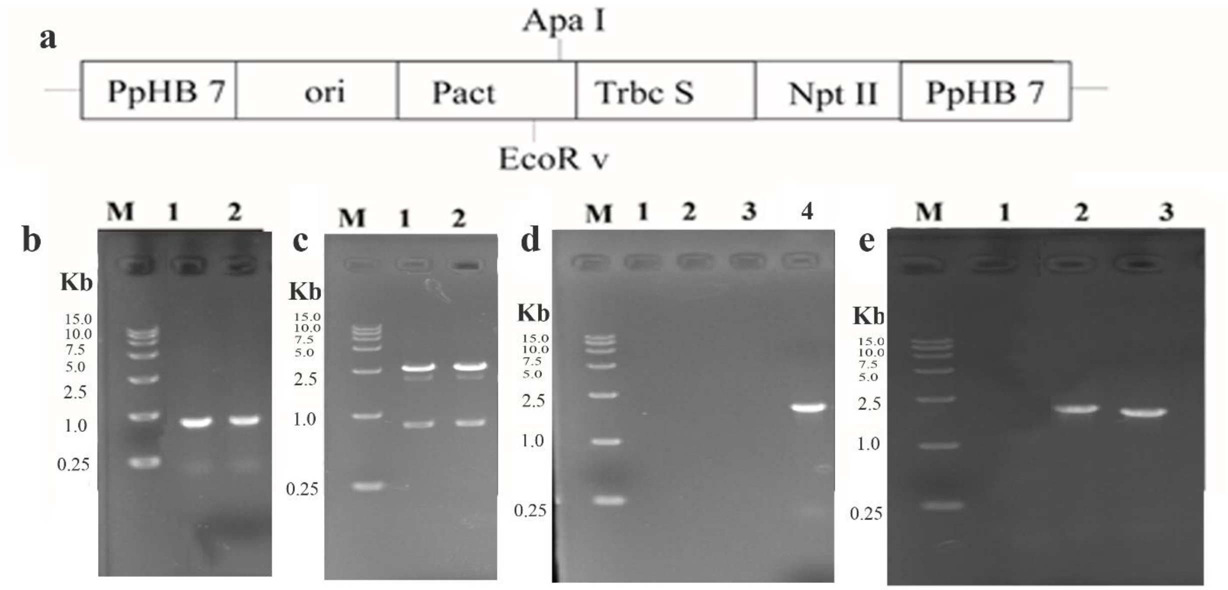

Supplement: Supplementary file 4 — Supplementary Material 4 [file 12870_2022_3892_MOESM4_ESM.pdf]
