# Supplementary material for: Physcomitrium patens CAD1 has distinct roles in growth and resistance to biotic stress
Source: BMC Plant Biol. 2022 Nov 8;22:518. doi: 10.1186/s12870-022-03892-3 (PMC9641914; doi:10.1186/s12870-022-03892-3)

Additional file 6 DFRC monomers from **a** *P. patens*, **b** *Arabidopsis* stem, **c** bamboo willow (*Salix salicaceae*) by GC-MS. **d** GC-MS Electrospray ionization MS/MS, quadrupole time-off light spectra of peaks G, S, and H monomers.

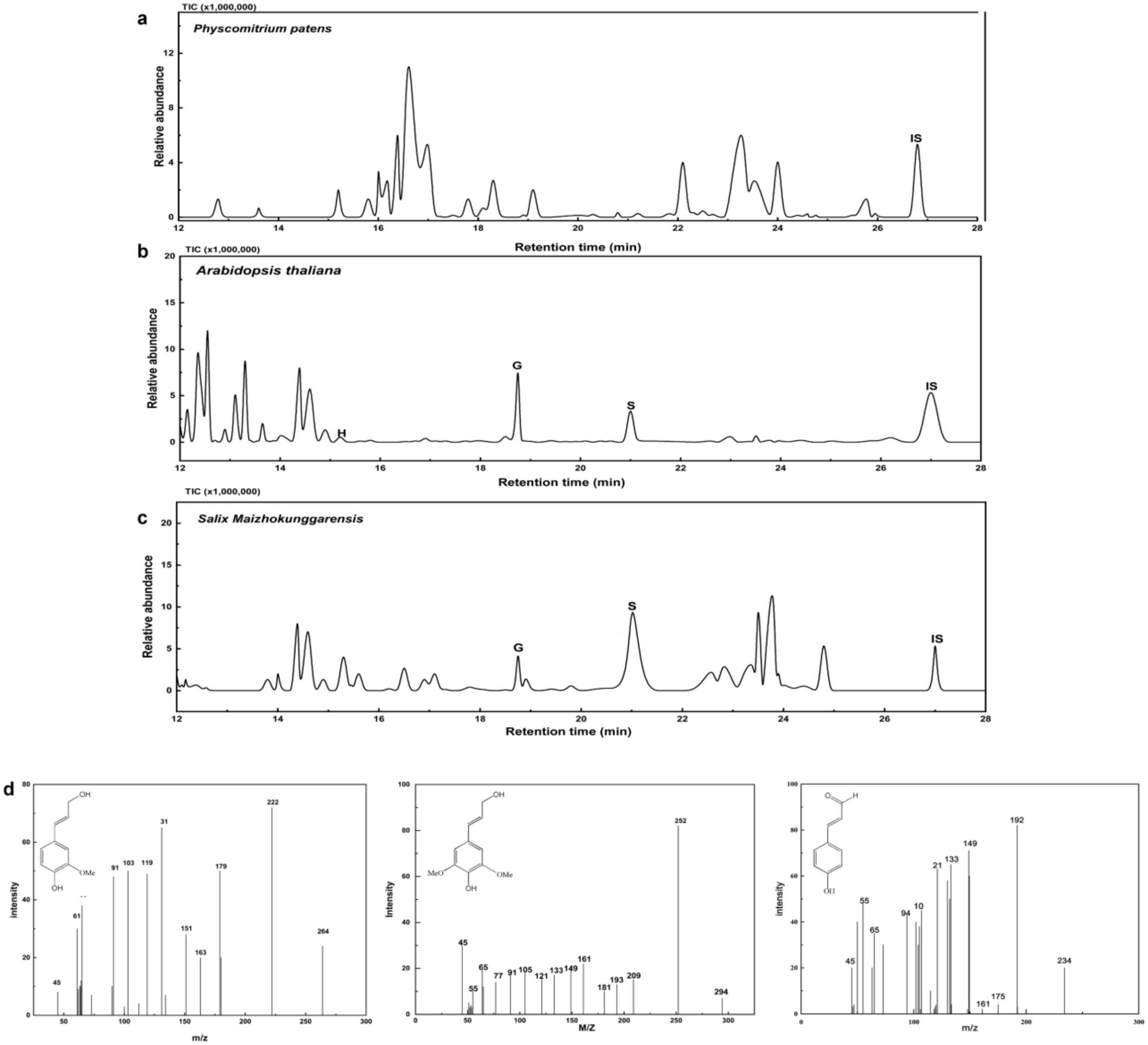

Supplement: Supplementary file 6 — Supplementary Material 6 [file 12870_2022_3892_MOESM6_ESM.pdf]
