# Supplementary material for: Physcomitrium patens CAD1 has distinct roles in growth and resistance to biotic stress
Source: BMC Plant Biol. 2022 Nov 8;22:518. doi: 10.1186/s12870-022-03892-3 (PMC9641914; doi:10.1186/s12870-022-03892-3)

Additional file 7 The compounds at the retention time of 30.79 min, 33.74 min, and 34.74 min were scanned at full wavelength by a diode array detection system.

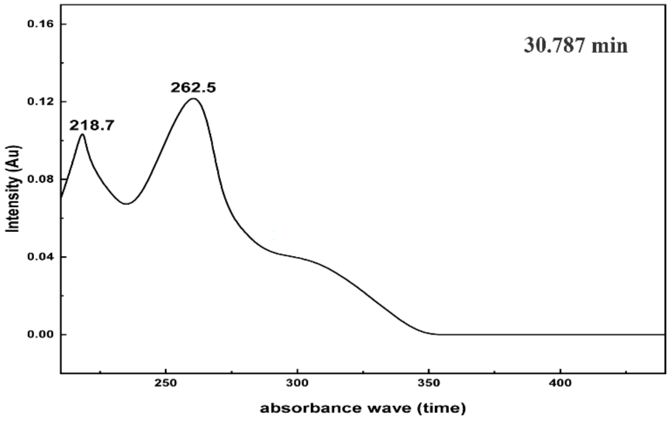

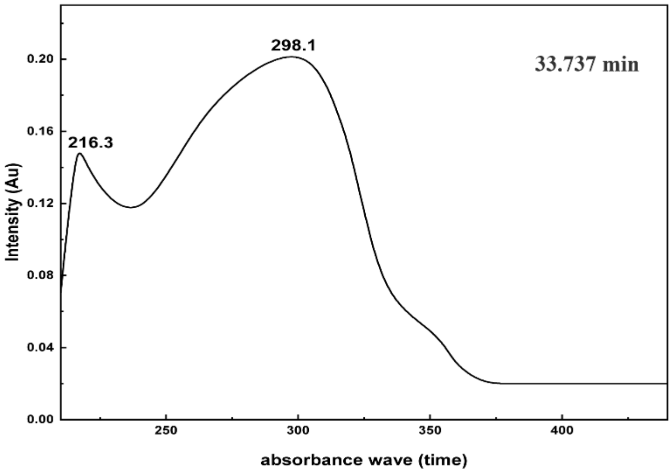

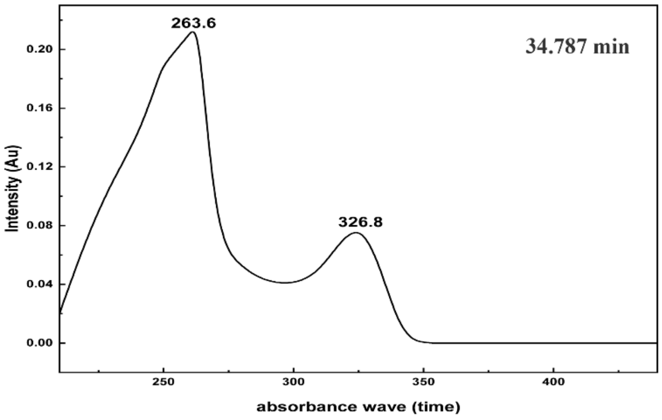

Supplement: Supplementary file 7 — Supplementary Material 7 [file 12870_2022_3892_MOESM7_ESM.pdf]
