# Supplementary material for: Physcomitrium patens CAD1 has distinct roles in growth and resistance to biotic stress
Source: BMC Plant Biol. 2022 Nov 8;22:518. doi: 10.1186/s12870-022-03892-3 (PMC9641914; doi:10.1186/s12870-022-03892-3)

Additional file 9 The transcripts of gene family encoded enzymes involved in the lignin biosynthesis in wild type and transformants. All data are presented as means ± SD and analyzed using one-way ANOVA. Different letters indicate significance in the expression levels at the 0.05 level.

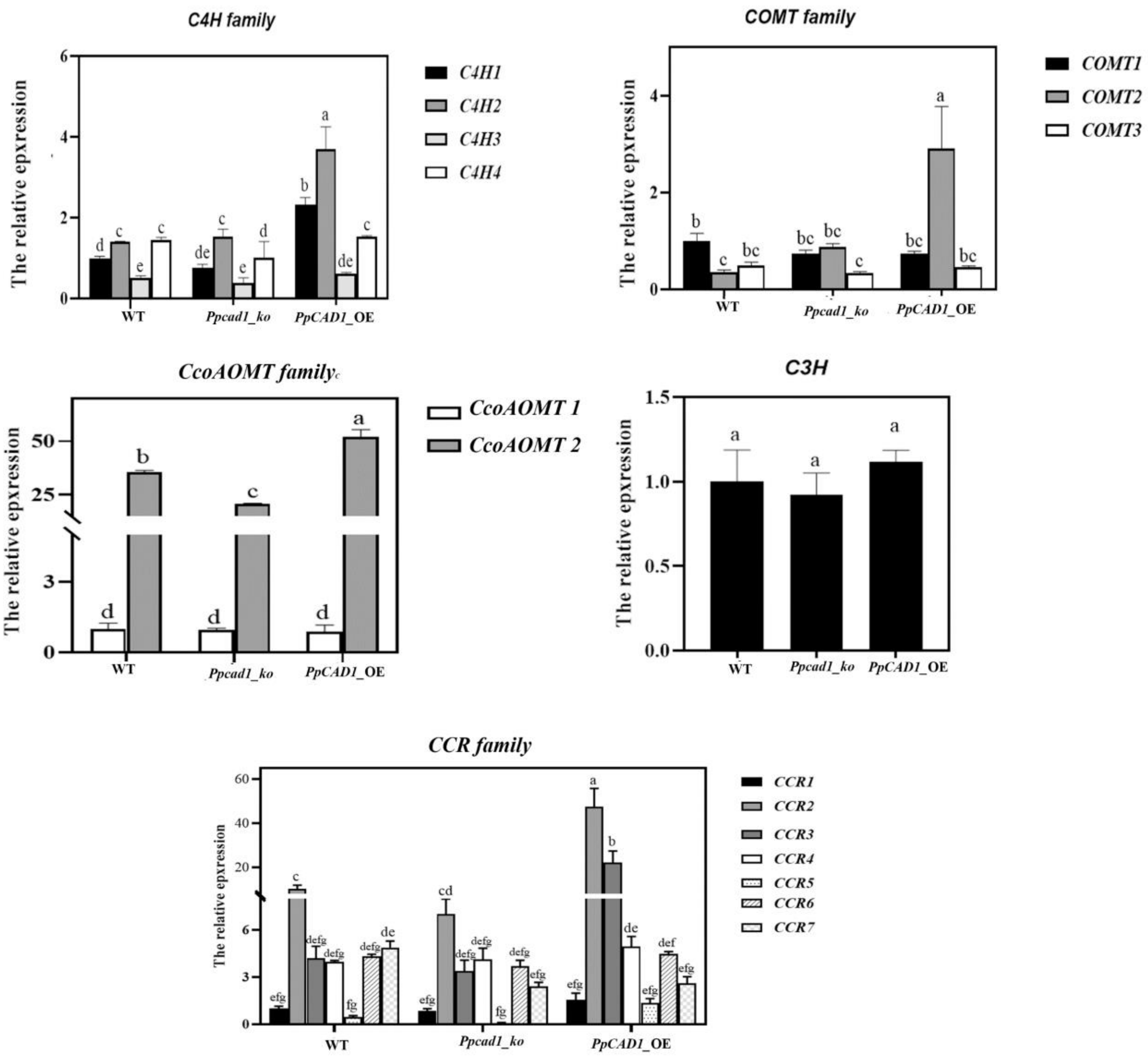

Supplement: Supplementary file 9 — Supplementary Material 9 [file 12870_2022_3892_MOESM9_ESM.pdf]
